# Large-scale analysis of macromolecular crowding effects on protein aggregation using a reconstituted cell-free translation system

**DOI:** 10.3389/fmicb.2015.01113

**Published:** 2015-10-08

**Authors:** Tatsuya Niwa, Ryota Sugimoto, Lisa Watanabe, Shugo Nakamura, Takuya Ueda, Hideki Taguchi

**Affiliations:** ^1^Department of Biomolecular Engineering, Graduate School of Bioscience and Biotechnology, Tokyo Institute of TechnologyYokohama, Japan; ^2^Department of Biotechnology, Graduate School of Agricultural and Life Sciences, The University of TokyoTokyo, Japan; ^3^Department of Computational Biology and Medical Sciences, Graduate School of Frontier Sciences, The University of TokyoChiba, Japan

**Keywords:** protein aggregation, protein folding, cell-free translation system, macromolecular crowding, large-scale analysis

## Abstract

Proteins must fold into their native structures in the crowded cellular environment, to perform their functions. Although such macromolecular crowding has been considered to affect the folding properties of proteins, large-scale experimental data have so far been lacking. Here, we individually translated 142 *Escherichia coli* cytoplasmic proteins using a reconstituted cell-free translation system in the presence of macromolecular crowding reagents (MCRs), Ficoll 70 or dextran 70, and evaluated the aggregation propensities of 142 proteins. The results showed that the MCR effects varied depending on the proteins, although the degree of these effects was modest. Statistical analyses suggested that structural parameters were involved in the effects of the MCRs. Our dataset provides a valuable resource to understand protein folding and aggregation inside cells.

To clarify the principles of the protein aggregation and the properties associated with it, we conducted a comprehensive analysis of protein aggregation under the completely chaperone-free condition by using a *Escherichia coli* reconstituted cell-free translation system (Niwa et al., 2009). In this analysis, thousands of bacterial proteins were expressed separately, and their aggregation propensities were evaluated by using a centrifugation-based method. Statistical analyses revealed significant insights concerning protein aggregation (Niwa et al., 2009).

In the previous analysis, the aggregation propensity was evaluated in a diluted solution, in which the protein concentration was at most 1–2 mg/mL (Shimizu et al., 2001, 2005; Niwa et al., 2009). However, the intracellular environment is much more crowded with macromolecules such as proteins and nucleic acids, and such an environment has been thought to affect the protein folding properties and the aggregation propensity (Zhou et al., 2008; Zhou, 2013). The effect of macromolecular crowding on protein folding and aggregation has been studied extensively, from both theoretical and experimental viewpoints, for decades (Zhou et al., 2008; Elcock, 2010; Gershenson and Gierasch, 2011; Zhou, 2013), and some studies suggested that the macromolecular crowding effects increase the intermolecular interactions mainly by its excluded volume effect, and hence facilitate the aggregation of some proteins (van den Berg et al., 1999; Munishkina et al., 2004). In contrast, other studies predicted that the crowding effects increase the stability of the native state and tend to bias proteins toward the native structure, although the effect on the stability was suggested to be modest (Cheung et al., 2005; Christiansen et al., 2010; Hong and Gierasch, 2010; Mittal and Best, 2010; Wang et al., 2010; Gershenson and Gierasch, 2011). However, in either case, these studies were limited to the experiments with a small number of model substrates or theoretical approaches.

To gain insight into the effects of macromolecular crowding on protein folding and aggregation and confirm these theories, we performed a large-scale analysis of the macromolecular crowding effects with a variety of proteins, by attempting the “*in vitro* proteome” approach reported previously (Niwa et al., 2009, 2012. By using a reconstituted cell-free translation system (Shimizu et al., 2001, 2005), we can easily evaluate the macromolecular crowding effects during the translation reaction for various kinds of proteins. In this analysis, we chose two macromolecular crowding reagents (MCRs), Ficoll 70, and dextran 70, because both two MCRs are hydrophilic polysaccharide and expected to have low interaction in specific amino acid side chains. Hence, the effects of these two MCRs can be thought to be mainly attributed to its excluded volume effect without any significant inhibition of expression reactions by the cell-free translation system (Zhou et al., 2008). In fact, we tried to use polyethylene glycol (PEG) 3350 as another MCR, but we could not evaluate its effect because the presence of PEG 3350 almost entirely abolished the protein expression by the cell-free translation system.

The method for the evaluation followed the previous comprehensive analyses of protein aggregation (see Materials and Methods). The measurement error of the solubility in the presence of the MCRs was about ±10%, which is nearly equal to that in the absence of MCRs, as reported previously (Niwa et al., 2009).

We performed this experiment for 150 *E. coli* proteins under three conditions: no addition of MCRs, Ficoll-added, and dextran-added conditions. These 150 proteins were chosen at random among the proteins that were annotated as cytoplasmic proteins and whose aggregation propensities were evaluated in the previous comprehensive analysis (Niwa et al., 2009). Among the tested proteins, 142 proteins were quantified under the three conditions. All obtained data are shown in Supplementary Table S1 in the dataset, which is available at figshare repository^1^. The distributions of the solubilities under the Ficoll- and dextran-added conditions were similar to that in the absence of MCRs (**Figure 1A**). This result suggested that Ficoll and dextran do not exert strong effects on the overall aggregation propensity. However, the distribution of the solubility changes under the dextran-added conditions was slightly biased toward a higher level, suggesting that dextran tends to act to prevent protein aggregation (**Figure 1B**). Furthermore, the solubility changes by Ficoll or dextran were widely distributed between -50 and +50%, suggesting that the MCRs could act both positively and negatively on aggregate formation. In other words, the degree or direction of the effect of the MCRs on the aggregation propensity depends on the properties of the proteins. Moreover, the solubility changes by Ficoll correlated well with those by dextran (**Figure 1C**), indicating the similar effects of the two MCRs on the aggregation propensity.

**FIGURE 1:**
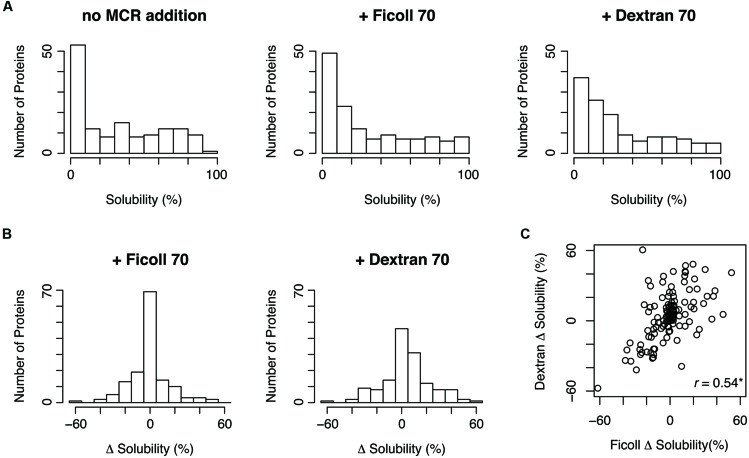
**Solubility distributions in the presence of macromolecular crowding reagents (MCRs) and the solubility changes by MCRs. (A)** Distribution of the solubilities in the absence and the presence of Ficoll 70 or dextran 70. **(B)** Distribution of the solubility changes by Ficoll 70 or dextran 70. The solubility change was defined by subtracting the solubility in the absence of MCRs from the solubility in the presence of MCRs. **(C)** Scatter plot of the solubility changes by Ficoll 70 and dextran 70. The Pearson’s correlation coefficient was 0.54 (^∗^*p* < 0.05).

As expected, the addition of both two MCRs did not cause drastic changes in the synthetic yield of the cell-free translation system. Furthermore, the change of the synthetic yield did not show a significant correlation with the solubility changes, suggesting that the effects of the changes in the synthetic yield on the solubility changes by the MCRs were small.

To determine which properties were related to the effects of the MCRs, we compared the solubility changes by MCRs and the physicochemical properties, such as molecular weight and isoelectric point. Although the molecular weight did not correlate with the solubility change, the solubility change by dextran positively correlated with the isoelectric point (**Figure 2A**). Moreover, the net charge, calculated by the number of charged amino acid residues, also correlated with the solubility change by dextran. These results suggested that dextran tends to act as an aggregation inhibitor for positively charged proteins. Concerning the properties derived from the primary sequence, we compared the solubility changes with the content ratios of the four amino acid groups, classified according to their properties. However, no obvious correlation was observed in the ratio of negatively charged (Asp and Glu), positively charged (Lys, Arg, and His), aromatic (Phe, Tyr, and Trp), or hydrophobic (Val, Leu, and Ile) amino acids.

**FIGURE 2:**
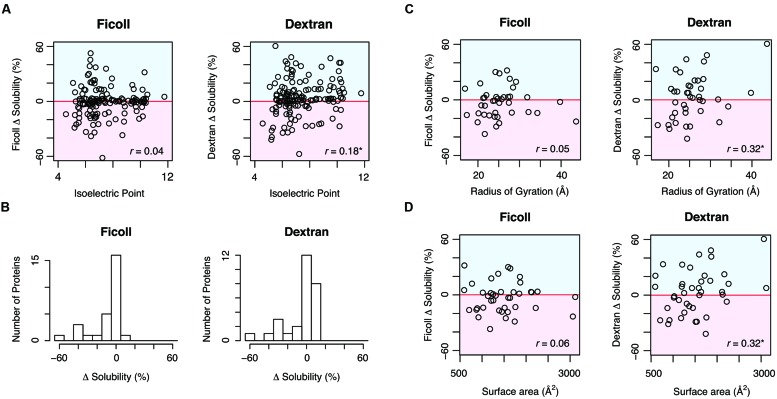
**Comparison between the solubility change by MCRs and the properties relevant to the primary amino acid sequences and structural features. (A)** Comparison between the calculated isoelectric point and the solubility change by Ficoll 70 and dextran 70. The Pearson’s correlation coefficients were 0.04 (*p* = 0.65) for Ficoll 70 and 0.18 (^∗^*p* < 0.05) for dextran 70. **(B)** Distribution of the solubility changes by MCRs among the proteins with aggregation-prone SCOP folds, observed in the previous report. The SCOP folds were annotated with the SUPERFAMILY database. The number of the proteins with aggregation-prone SCOP folds was 28. **(C)** Scatter plot of the radius of gyration and the solubility changes by MCRs. The Pearson’s correlation coefficients were 0.05 (*p* = 0.76) for Ficoll 70 and 0.32 (^∗^*p* < 0.05) for dextran 70. **(D)** Scatter plot of the main chain surface area and the solubility changes by MCRs. The Pearson’s correlation coefficients were 0.06 (*p* = 0.71) for Ficoll 70 and 0.32 (^∗^*p* < 0.05) for dextran 70.

Most proteins adopt a unique tertiary structure defined by the amino acid sequence, and a wide variety of structures exist. To compare the structural properties of proteins with the effects of the MCRs, we used the structural classification of proteins (SCOP) database (Murzin et al., 1995). As reported previously, the SCOP fold seems to have a formidable influence on the aggregation propensity, and proteins with specific folds have a strong tendency to form aggregates (Niwa et al., 2009). We then extracted the proteins with aggregation-prone folds, and investigated the distribution of their solubility changes by the MCRs. All annotations of the SCOP folds for tested proteins are listed in Supplementary Table S2 in the dataset^1^, and the aggregation-prone folds in the previous report (Niwa et al., 2009) were as follows; c37: P-loop containing nucleoside triphosphate hydrolases, a4: DNA/RNA-binding 3-helical bundle, c1: TIM β/α-barrel, c3: FAD/NAD(P)-binding domain, c55: Ribonuclease H-like motif, and c94: Periplasmic binding protein-like II. The histograms of the solubility changes for the proteins with the aggregation-prone folds revealed strong biases toward lower solubility, indicating that the MCRs tended to enhance the aggregate formation of these proteins (**Figure 2B**).

To compare further structural features, we constructed structural models for 41 proteins, by using a template-based modeling method. The structural templates used for the modeling are listed in Supplementary Table S3 in the dataset^2^. Comparisons of the radius of gyration and the surface area of the amino acid main chains showed that both parameters positively correlated with the solubility change by dextran (**Figures 2C,D**). In addition, we compared the solubility change by the MCRs and the relative contact order, which is considered to be related to protein folding (Plaxco et al., 1998). Although the contact order negatively correlated with the solubility change by dextran, the correlation between them was not statistically significant.

The data obtained from this study suggested that the macromolecular crowding effect enhances aggregate formation for some proteins and prevents it for others. Previous experimental and theoretical studies suggested that the macromolecular crowding effects are often quite complicated, and particularly difficult to understand quantitatively (Zhou et al., 2008; Elcock, 2010; Zhou, 2013). The results obtained here seem to reflect this complexity of the macromolecular crowding effects on protein folding and aggregation. In addition, some studies suggested that the effect of macromolecular crowding on protein folding is modest (Zhou et al., 2008; Mittal and Best, 2010; Zhou, 2013). Our data seem to be in agreement with these ideas, because the effects of macromolecular crowding were not strong, in comparison with the influences of molecular chaperones reported previously (**Figure 1A**; Niwa et al., 2012). Although our statistical analyses gave some insights for understanding the macromolecular crowding effects as described above, their influences on protein folding and aggregation are quite complicated and further detailed analysis are needed. Our dataset obtained from the “*in vitro* proteome” approach has great potential, as a valuable dataset that will contribute to further understanding of the effects of macromolecular crowding and protein folding inside cells.

## Materials and Methods

### Method for the Evaluation of the Aggregation Propensity

The method for the evaluation of the aggregation propensity followed those used in previous comprehensive analysis (Niwa et al., 2009). The template DNA for expression by the cell-free translation system was amplified from an *E. coli* ORF library [ASKA library (Kitagawa et al., 2005; Riley et al., 2006)] by PCR, as described previously (Niwa et al., 2009). The transcription-translation-coupled expression was conducted by a reconstituted cell-free translation system [PURE system (Shimizu et al., 2001, 2005)] at 37°C for 1 h. For detection, L-[^35^S]-methionine was added to the PURE system. Ficoll 70 (GE Healthcare) or dextran 70 (Sigma–Aldrich) was also included at the concentration of 80 mg/ml in the reaction, to evaluate the effects of MCRs. After the expression, an aliquot was withdrawn as the total fraction, and the remainder was centrifuged at 20,000 × *g* for 30 min. The total and supernatant fractions were separated by SDS-PAGE, and the band intensities were quantified by autoradiography (FLA7000 image analyzer and Multi Gauge software, Fujifilm). The ratio of the supernatant to the total protein was defined as the solubility, as referred to as the index of aggregation propensity.

### Data Analysis

The molecular weight, amino acid content, and net charge were calculated from the amino acid sequences obtained from GenoBase^3^ (Kitagawa et al., 2005; Riley et al., 2006). Estimation of pI values was conducted with a web tool^4^ (Sillero and Maldonado, 2006). The SCOP (Murzin et al., 1995) classification was obtained from the dataset distributed by GenoBase. The SCOP fold annotation in GenoBase was based on the SUPERFAMILY database (Madera et al., 2004). The SCOP folds annotated as aggregation-prone folds were as follows; c37: P-loop containing nucleoside triphosphate hydrolases, a4: DNA/RNA-binding 3-helical bundle, c1: TIM β/α-barrel, c3: FAD/NAD(P)-binding domain, c55: Ribonuclease H-like motif, and c94: Periplasmic binding protein-like II (Niwa et al., 2009). The modeled structures were obtained from the database by Zhang’s group^5^ or modeled by the MODELER program^6^ (Eswar et al., 2006). Among the 41 modeled structures, 28 were selected from Zhang’s database with the following criteria: >80% template identity, >80% template coverage, and >0.7 TM-score to the template. The remaining 13 structures were modeled by MODELER with the template PDBs determined by a PSI-BLAST search with the following criteria: >80% template identity and >80% template coverage. The radius of gyration and the relative contact order were calculated by using in-house developed scripts. Surface area was calculated with the NACCESS software^7^ (Hubbard and Thornton, 1993). All statistical tests were conducted with the R software^8^

## Conflict of Interest Statement

The authors declare that the research was conducted in the absence of any commercial or financial relationships that could be construed as a potential conflict of interest.

## Footnotes

1 http://dx.doi.org/10.6084/m9.figshare.1495333

2 http://dx.doi.org/10.6084/m9.figshare.1495333

3 http://ecoli.naist.jp/GB/

4 http://isoelectric.ovh.org/

5 http://zhanglab.ccmb.med.umich.edu/QUARK/ecoli2/

6 https://salilab.org/modeller/

7 http://www.bioinf.manchester.ac.uk/naccess/

8 http://www.r-project.org/
